# A maximum-likelihood approach for building cell-type trees by lifting

**DOI:** 10.1186/s12864-015-2297-3

**Published:** 2016-01-11

**Authors:** Nishanth Ulhas Nair, Laura Hunter, Mingfu Shao, Paulina Grnarova, Yu Lin, Philipp Bucher, Bernard M. E. Moret

**Affiliations:** School of Computer and Communication Sciences, École Polytechnique Fédérale de Lausanne (EPFL), EPFL IC IIF LCBB, INJ 211 (Batiment INJ), Station 14, Lausanne, CH-1015 Switzerland; Computer Science Department, Stanford University, Stanford, USA; Department of Computer Science and Engineering, University of California, San Diego, San Diego USA; School of Life Sciences, École Polytechnique Fédérale de Lausanne (EPFL), Lausanne, Switzerland; Swiss Institute of Bioinformatics, Lausanne, Switzerland

**Keywords:** Cell-type trees, Histone modifications, Epigenomics, Phylogeny, Evolution, Cell-differentiation

## Abstract

**Background:**

In cell differentiation, a less specialized cell differentiates into a more specialized one, even though all cells in one organism have (almost) the same genome. Epigenetic factors such as histone modifications are known to play a significant role in cell differentiation. We previously introduce cell-type trees to represent the differentiation of cells into more specialized types, a representation that partakes of both ontogeny and phylogeny.

**Results:**

We propose a maximum-likelihood (ML) approach to build cell-type trees and show that this ML approach outperforms our earlier distance-based and parsimony-based approaches. We then study the reconstruction of ancestral cell types; since both ancestral and derived cell types can coexist in adult organisms, we propose a lifting algorithm to infer internal nodes. We present results on our lifting algorithm obtained both through simulations and on real datasets.

**Conclusions:**

We show that our ML-based approach outperforms previously proposed techniques such as distance-based and parsimony-based methods. We show our lifting-based approach works well on both simulated and real data.

## Background

Cell differentiation is the process by which a less specialized cell becomes a more specialized one; it often proceeds in a hierarchical manner, with totipotent cells sequentially committing to fates of more restricted developmental potential [1, 2]. Epigenetic and transcription factors play a significant role in cell differentiation [3–5], therefore motivating a study of epigenetic changes across different cell types.

Arendt [6] proposed a sister-cell-type model for the hierarchical relationship between cell types. In this model [6], “novel cell types arise in pairs (sister cell types) from an ancestral cell type through sub-specialization” [2]. Under this model [6], the evolutionary relatedness of cell types is expected to be congruent with the ontogenetic hierarchy of cellular differentiation, because the “development of the sister cell types is the same up to the last stages of differentiation” [2]. The authors claim that multifunctionality has been a general feature of ancient cell types and that, with “increasing specialization during evolution, these multiple functions were then distributed in a complementary manner to sister cell types” [6].

Evolution and cell differentiation share a number of attributes. First, as mentioned before, we know that cell differentiation transforms less specialized cell types into more specialized ones. Since this transformation is unidirectional, the paths of differentiation can be represented as a tree structure, much as is done with the phylogenetic trees used to represent evolutionary histories [7]. The similarity between the two extends further: cell types themselves have evolved into larger collections from more restricted collections in early ancestors: there are phylogenetic relationships among the various types of cells. Second, observed changes in the epigenetic state are inheritable, again much as mutations in the genome are (although, of course, through very different mechanisms and at very different scales); and finally, epigenetic traits are subject to stochastic changes. One major difference between evolution and cell differentiation is that functional changes in cell differentiation are primarily driven by programmed mutational events rather than by selection. However, the program of mutational events is itself the result of evolution, so that, as observed by [6], the cell differentiation tree often recapitulates the phylogeny of cell types.

We focus here on one important epigenetic mark—histone modification. Histones are proteins that package the DNA into nucleosomes [8]. These proteins are subjected to various types of chemical modifications, called histone modifications, such as methylation, acetylation, phosphorylation, ubiquitination, etc. These modifications alter their interactions with the DNA and thereby influence transcription and genomic function. Histone modifications have been found to vary across cell types and to play an important role in gene regulation [9]. Since histones are present in every 200 bp length of DNA, we need genome-wide high-throughput technologies to study the modifications of these proteins. ChIP-Seq is such a technology [10, 11]. The study of ChIP-Seq histone modification data can help us understand the role of histone modifications in developmental biology and cell differentiation [12].

The term “cell-type tree” was defined by our group to refer to a tree relationship between various cell types [13]. The nodes of this tree represent cell types while the edges represent directed differentiation/evolution events from one cell type to another. We know that the genome is consistent across cell types of the same individual and that it is also highly similar between individuals of the same species, but that epigenomic states of various regions of the genome differ across various cell types. These epigenomic states are believed to affect cell differentiation process through a complex interplay between histone modifications, DNA methylation, transcription factors, etc.

Kin et al. [2] recently constructed a cell-type tree using RNA-Seq data and a parsimony-based approach under assumptions very similar to ours, using the same term of “cell-type tree” to denote the “hypothetical tree-like relationship of cell types in ontogeny and phylogeny”. Liang et al. [14] recently developed a statistical model for cell differentiation and applied it to ENCODE and FANTOM RNA-Seq data. As in Kin et al. work [2], they found that the RNA-Seq data contain significant tree structures. In earlier work [7], we also calculated a statistical measure to show that the distances we computed are in fact representative of a tree. Thus multiple studies on different kinds of datasets—ChIP-Seq in our case, RNA-seq in the other two papers—support the tree-like relationship of cell types and underscore the usefulness of the cell-type tree (as noted in [2]). Prior to these genome-wide computational approaches, hierarchical developmental relationships among cell types were elucidated through a series of laborious experiments involving in vitro differentiation of cell types from various stem cells [15–17].

In cell differentiation, both ancestral and derived cell types can coexist within the body. Therefore, it becomes important to be able to infer which cell types should be treated as the ancestor, or parent, of another. Our earlier work [7, 13] focused on the use of distance-based and parsimony-based phylogenetic methods to infer the tree, not the ancestors. Here we propose an ML approach to the inference of cell-type trees on histone modification data and proceed to derive a new algorithm to infer the internal nodes by a process known as lifting. (Since both ancestral and derived cell types can coexist in the body, some of the node labels should be simply “lifted”—copied—into the parent node). To our knowledge, this is the first lifting approach used in the study of cell differentiation. We also provide simulations and tests on real data, indicating that our ML approach to the inference of cell-type trees outperforms distance-based and parsimony-based building approaches and that our lifting algorithm not only works well on simulations, but also gives biologically meaningful results.

## Methods

A histone-modification ChIP-Seq library contains ChIP-Seq data for one ChIP-Seq experiment. In our case, each library typically contains data for one histone modification for one replicate of a cell type.

**Fig. 1:**
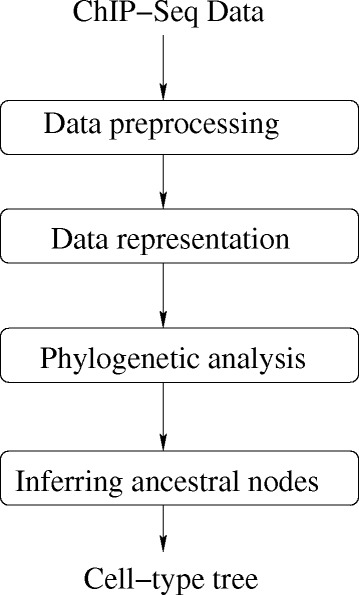
Flowchart for building cell-type trees using a maximum-likelihood framework. Data preprocessing: the mapped reads in the ChIP-Seq data are used to find peaks in the data. Data representation: peak data is converted to a binary matrix using windowing/overlap representation. Phylogenetic analysis: using distance-based, parsimony-based or maximum-likelihood-based phylogenetic approach. Inferring ancestral or internal nodes: we establish a parent-child relationship between the cell types (leaf data) using a process called lifting

Our approach to build cell-type trees using a ML framework is illustrated in Fig. 1. We explain the various steps below.

### Model of differentiation for histone marks

We use the model of [7], in which histone marks can be independently gained or lost in regions of the genome as cells differentiate from a less specialized type to a more specialized one. This independence assumption is consistent with practice in phylogenetic inference and enormously simplifies computations.

### Data preprocessing and data representation

ChIP-Seq data are converted into peak data using a peak-finder. The presence of a peak signifies the presence of a histone mark in that genomic region. We have used publicly available peak lists (from the ENCODE database) for our study.

To represent the peak data of each cell type, we use two different data representations.

1. **Windowing representation**: The genome is divided into bins of fixed size; if the bin contains at least one peak, we code it 1, otherwise we code it 0. The coding of each library is thus independent of that of any other library (a ChIP-Seq dataset and its representation are both called libraries). We used bins of 200 bp because 147 bp of DNA wrap around the histone and two histones are connected by linker DNA of about 50 bp; thus each bin approximates the presence or absence of just one histone modification.
2. **Overlap representation**: This representation takes into account all libraries at once. The aim is to find interesting regions in the genome based on peaks. Denote the *i*^*th*^ peak in library *n* as ${P^{n}_{i}}=\left [P^{n}_{\textit {iL}},P^{n}_{\textit {iR}}\right ]$, where $P^{n}_{\textit {iL}}$ and $P^{n}_{\textit {iR}}$ are the left and right endpoints (as basepair indices). Consider each peak as an interval on the genome (or on the line of real numbers) and build the interval graph defined by all peaks in all libraries. An interval graph has one vertex for each interval and an edge between two vertices whenever the two corresponding intervals overlap [13]. We simply want the connected components of the interval graph. We define an interval in the genome is an *interesting region* if and only if it corresponds to a connected component of the interval graph. More details on the overlap representation and an algorithm to identify interesting regions in linear time appear in [7].

The output of either the windowing representation or overlap representation is a string of ones and zeros to represent each data library. Both representations gave fairly similar results in earlier work [7], so in this study we chose the overlap representation, for its compactness.

### ML-based phylogenetic analysis

We use an ML-based approach to build cell-type trees on the overlap data representation, carrying out the inference with the RAxML tool [18]. We run RAxML on the binary data obtained using the overlap representation and obtain a cell-type tree. For our experiments, we used the GAMMA model of rate heterogeneity (BINGAMMA) and turned on the rapid bootstrapping option (set to 100).

### Inferring ancestral/internal nodes through lifting

We now describe an algorithm for inferring ancestral/internal nodes using a process called lifting, to establish a parent-child relationship between the various cell types (at the leaves). Lifting techniques have been used in the context of tree alignment problems [19]. We first infer a tree using RAxML; we then root the tree using prior biological knowledge. (One could root the tree by placing a root between two nodes of an unrooted tree connected by an edge). Denote by *T* the resulting rooted binary tree. We now run the lifting procedure on this tree *T*, so that they obey path constraints. The basic idea of the lifting procedure is to compute the likelihood of the tree after the lifting each possible leaf node (if its sibling is also a leaf) and subtract it from the likelihood of the unlifted tree, and then to actually lift the leaf with the highest probability if this difference is greater than some threshold. The old tree is now updated with the lifted tree and this procedure is continued till the lifting stops. The pseudocode for the lifting algorithm is given below.

#### Algorithm for lifting

1. Set *R*=*T*.
2. If number of leaves in *R* is less than or equal to 4, go to step 7.
3. Let $\mathbb {L}$ be the set of leaves whose sibling is also a leaf. For each leaf node $L \in \mathbb {L}$ we compute the likelihood/probability *P*(*L*) of lifting *L* using the following procedure.
  - Divide the tree *R* into two smaller trees according to *L*, ${R_{L}^{1}}$ and ${R_{L}^{2}}$ (see example in Fig. 2). ${R_{L}^{1}}$ is built by first lifting *L* to its parent and then removing *L* and its sibling (from *R*). ${R_{L}^{2}}$ is a small tree of 2 nodes, the earlier leaf node *L* which is connected to its sibling node (parent is *L*).

**Fig. 2:**
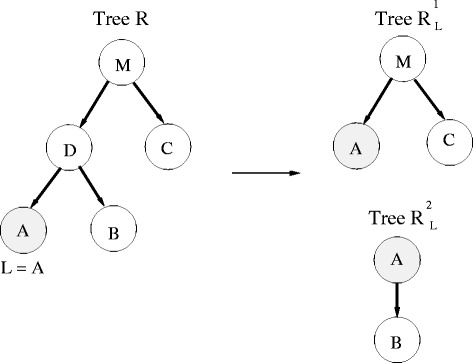
Example of lifting leaf node *A* (*L* in the algorithm) in tree *R*. Tree *R* is divided into two smaller trees ${R_{L}^{1}}$ and ${R_{L}^{2}}$ as described in the algorithm
  - Estimate the probabilities of ${R_{L}^{1}}$ and ${R_{L}^{2}}$ (branch lengths estimated using RAxML and probabilities calculated using built in methods in *R* software packages like *phangorn* [20]). The total probability is the product of these two probabilities.
4. Compute the best lift $L^{*} = \arg \max _{L \in \mathbb {L}} P(L)$.
5. Let *W*=*log*(*P*(*L*^∗^))+*αlog*(*K*)−*log*(*P*(*R*)), where *K* is the length of the data representation sequence, *α* a user-defined, real-valued constant, *P*(*R*) is the likelihood of tree *R*. *W* plays a role similar to a BIC criterion [21].
6. If *W*>0, we lift, update the tree $R = R_{L^{*}}^{1}$, and mark the corresponding edge (parent of *L*^∗^ and *L*^∗^) in *T* as lifted; we then return to step 2.
7. Output *T* and stop.

The output tree *T* is the desired cell-type tree with labelled ancestral nodes. We terminate the algorithm when the number of leaves is four or less since we use RAxML.

## Results and discussion

We show the results on both real and simulated data.

### Using real data

In an earlier work [7], we had shown the usefulness of using cell-type trees on different histone marks from the ENCODE project database: H3K4me3, H3K27me3, H3K4me1, H3K9me3, and H3K27ac. For testing the lifting algorithm, it is desirable to have as many samples as possible. We thus focus in this study on histone modification H3K4me3, using ChIP-Seq data for human (hg19) from the University of Washington ENCODE group [22, 23]. H3K4me3 has been assayed in the largest number of cell types and is usually associated with gene activation [24]. Table 1 gives the list of the 37 cell types for which we gathered H3K4me3 data. The cells are classified into groups based on cell type or tissue origin. (Keratinocytes (NHEK) is included in the Epithelial group). For human Embryonic Stem Cells (hESC) we have data for a differentiation time course in cell culture (day 0, 2, 5, 9, 14), so we shall use hESC T2 to mean data for hESC cells on day 2. We use only one replicate per cell type for this work.

**Table 1:** Cell types, short description, and general group for H3K4me3 data. For details see the ENCODE website [23]

Cell name	Short description	Group
AG04449	fetal buttock/thigh fibroblast	Fibroblast
AG04450	fetal lung fibroblast	Fibroblast
AG09319	gum tissue fibroblasts	Fibroblast
AoAF	aortic adventitial fibroblast cells	Fibroblast
BJ	skin fibroblast	Fibroblast
CD14	Monocytes-CD14+ from human leukapheresis production	Blood
CD20	B cells replicate	Blood
hESC	undifferentiated embryonic stem cells	hESC
HAc	astrocytes-cerebellar	Astrocytes
HAsp	astrocytes spinal cord	Astrocytes
HBMEC	brain microvascular endothelial cells	Endothelial
HCFaa	cardiac fibroblasts- adult atrial	Fibroblast
HCF	cardiac fibroblasts	Fibroblast
HCM	cardiac myocytes	Myocytes
HCPEpiC	choroid plexus epithelial cells	Epithelial
HEEpiC	esophageal epithelial cells	Epithelial
HFF	foreskin fibroblast	Fibroblast
HFF MyC	foreskin fibroblast cells expressing canine cMyc	Fibroblast
HMEC	mammary epithelial cells	Epithelial
HPAF	pulmonary artery fibroblasts	Fibroblast
HPF	pulmonary fibroblasts isolated from lung tissue	Fibroblast
HRE	renal epithelial cells	Epithelial
HRPEpiC	retinal pigment epithelial cells	Epithelial
HUVEC	umbilical vein endothelial cells	Endothelial
HVMF	villous mesenchymal fibroblast cells	Fibroblast
NHDF Neo	neonatal dermal fibroblasts	Fibroblast
NHEK	epidermal keratinocytes	Epithelial
NHLF	lung fibroblasts	Fibroblast
RPTEC	renal proximal tubule epithelial cells	Epithelial
SAEC	small airway epithelial cells	Epithelial
SKMC	skeletal muscle cells	Skeletal muscle
WI 38	embryonic lung fibroblast cells	Fibroblast

**Fig. 3:**
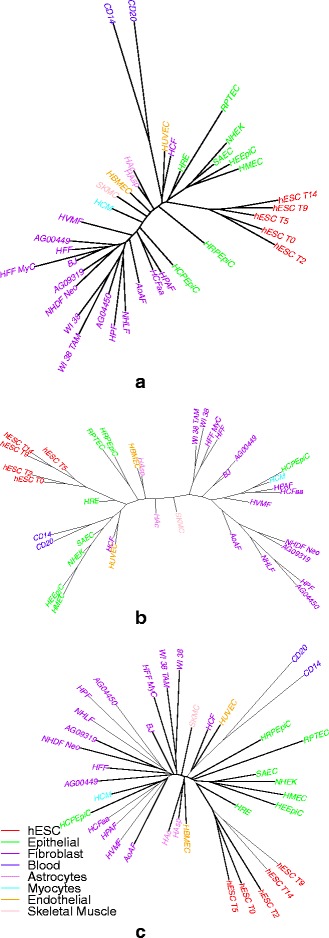
Compares cell-type trees obtained on H3K4me3 data (only one replicate) using two different methods on the overlap representation: (**a**) using a maximum-likelihood based approach, (**b**) using a parsimony-based approach, (**c**) using a distance-based approach

We use the ENCODE peaks as input to our program. We convert the input data into 1s and 0s using the overlap representation. We then use RAxML for getting a maximum likelihood based tree. In this first step, we then compare our results with those obtained with a distance-based approach (neighbor-joining [25]) and a parsimony-based approach (TNT [26]), as explained in [7]. Figure 3 summarizes the results, using color codes for the major groupings of Table 1. In order to quantify the quality of the groupings, we compute the total number of cell types in a subtree that belong to one group. Since our groups are based on cell type only, there could be many subdivisions possible within each group, therefore we choose the two largest such subtrees available for each group such that each subtree contains only the leaf nodes of that group.

Table 2 shows the results for the ML-based, parsimony-based, and distance-based methods. Cell-type trees were built without inferring ancestral/internal nodes (no lifting). The ML approach gives significantly better clades than the other two. For example, the subtrees containing Fibroblasts and Epithelial cell types are greatly improved. Of the 16 Fibroblast cell types that we considered, the ML method grouped 15 in the largest clade, a major improvement from the 6 grouped by the distance-based and parsimony-based approaches. For Epithelial cell types the improvement is from 4 to 6, out of 8 cell types in total.

**Table 2:** Groupings for cell-type trees on H3K4me3 data

	hESC	Epithelial	Fibroblast	Blood	Astrocytes	Myocytes	Endothelial	Skeletal Muscle
	(5)	(8)	(16)	(2)	(2)	(1)	(2)	(1)
D	5,0	4,1	6,3	2,0	2,0	1,0	1,1	1,0
P	5,0	4,2	6,4	2,0	1,1	1,0	1,1	1,0
ML	5,0	6,1	15,1	2,0	2,0	1,0	1,1	1,0

Second to ninth columns show the number of cell types (of the same group) belonging to the largest and second-largest clades; the total number of cell types of that group is in the top row. Rows correspond to various methods. Overlap representation is used. ML — maximum-likelihood-based approach, P — parsimony-based approach, D — distance-based approach

**Fig. 4:**
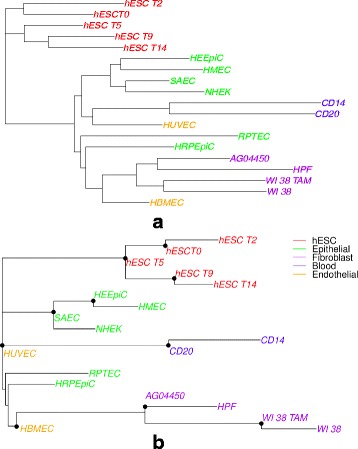
Cell-type trees obtained on H3K4me3 data on a set of 19 cell types: (**a**) before lifting, (**b**) after lifting

We now consider a set of 19 cell types to evaluate the lifting algorithm (on H3K4me3 data) to infer ancestral nodes. The cell types include the hESC time course of 5 days (day 0, 2, 5, 9, 14), HUVEC (umbilical vein endothelial cells), HBMEC (brain microvascular endothelial cells), WI 38 (embryonic lung fibroblast cells), AG04450 (fetal lung fibroblast), HPF (pulmonary fibroblasts isolated from lung tissue). As explained in [7], we expect the following developmental pathways to occur one after the other in time during development: (1) hESC from days 0 to 14; (2) hESC to HUVEC to HBMEC; (3) hESC to WI38 to AG04550 to HPF. We now use the overlap representation and RAxML to get the cell-type tree. The tree was now rooted at the common ancestor of all the embryonic stem cells at different days (see Fig. 4a). The lifting algorithm is then used. The *α* parameter is set high enough such that lifting takes place as much as possible, the larger the value of *α* the more the number of lifts. The results are shown in Fig. 4b. We find that there is a path from hESC to HUVEC to HBMEC as expected. We also find a path from hESC to AG04550 to HPF. However we see that WI38 could not be lifted before AG04450. When we look at the data for hESC data, we see that embryonic stem cells on day 5 is an ancestor (internal node) to day 9, and day 9 is an ancestor to day 14; and we also see day 0 is an ancestor to day 2. However day 5 is incorrectly lifted above day 0. On the other hand, the fact that day 2 ends up on a side branch of the tree is not surprising in view of our previous observation [7] that day 2 shows overall increased divergence in histone modification. This most likely reflects a temporary non-specific response to the growth factor cocktail that was applied on day 0 to activate a mesoderm developmental pathway.

We repeated these experiments by picking a random number of columns from the overlap representation (sampling without replacement) and we found that our results are quite consistent, if we choose 50,000 or even only 10,000 columns (out of a total of 200,152 columns).

### Using simulated data

In this paper, we use simulated studies to compare the quality of our maximum-likelihood approach and lifting algorithm. We randomly generate a set of rooted binary trees with a fixed number of leaves — containing either 12, 50, and 100 leaf nodes (random trees created using “ape” library in *R* [27]). Ten trees for each kind of tree (12, 50, or 100 leaf tree) are generated. Each of these trees then had their edges marked (randomly) as lifted (signifying lift from child to parent in that edge) with some probability. This is the true tree. Data representation length (*K*) of 1000 bits (1 or 0) for each leaf node was then simulated using each tree (used phangorn library in *R*).

A RAxML tree is generated on this leaf data and then this tree is given as input to the lifting program (for *α*=0.1). For the output tree, each edge which contains a lift from child to parent, is marked as lifted. This way we can compare the edges in the output tree with the true tree. Edges marked as lifted in the true tree should be shown as lifted in the output tree. Based on this, for each kind of trees (12, 50 or 100 leaf tree), we find the total number of true positives (TP), false positives (FP), true negatives (TN), false negatives (FN). We measure the following statistics [28] from this information:

1. True positive rate $TPR = \frac {TP}{TP+FN}$.
2. False positive rate $FPR = \frac {FP}{FP+TN}$.
3. F-score or *F*_1_-score $F = \frac {2TP}{2TP + FP + FN}$
4. Accuracy $ACC = \frac {TP+TN}{TP+TN+FP+FN}$

We also compute the Robinson-Foulds metric or *RF* distance [29] which computes the distance between the true tree and the unlifted tree we get after applying RAxML.

The results are shown in Table 3. We see that we get high true positive rate, low false positive rate, a reasonably high F-score, and an accuracy of above 90 %. These results thus show that our lifting approach produces good quality results for trees which are both small and large. Since we expect cell-type trees to be build on only a few dozen cell types (since there are about 200 cell types in humans and we don’t have data for many of these), we feel the range of tree sizes that we have considered in the simulation study is sufficient. We also get low *RF* distances for both 12-leaf and 50-leaf trees. The slightly higher RF distances we get for 100-leaf tree is because the data representation of 1000 is not large enough for bigger trees.

**Table 3:** Statistics for trees with fixed number of leaf nodes

	12-leaf	50-leaf	100-leaf
*TPR*	0.750	0.736	0.789
*FPR*	0.070	0.064	0.036
*F*	0.677	0.629	0.748
*ACC*	0.906	0.917	0.946
*RF*	1.3	5.900	12.20

We simulate 10 random trees (data representation length is 1000) for each of kind of tree (12, 50 or 100 leaf tree) and ran the lifting algorithm (for *α*=0.1). We then calculated the following statistics shown in the table: True positive rate (*TPR*), False positive rate (*FPR*), F-score or *F*_1_-score (*F*), Accuracy (*ACC*), *RF* distance (*RF*)

To show the robustness of our approach, we repeat the above experiments (for 12-leaf trees) by varying the length of data representation (*K*) per cell type. We use the following values — 500, 1000, and 5000 bits for each node. Various statistics are calculated by fixing the *α* threshold to 0.1. The results are shown in Table 4. We find that the different statistics are stable across different data representation lengths and the accuracy is around 90 %, and the statistics (like accuracy) improve with larger *K* which is expected. The *RF* distance is around 1 for different values of *K*. This shows that the threshold *α* is stable for a large variation of data representation lengths. We note that the choice of threshold *α* is dependent on the dataset used. The larger the value of *α* the greater the chances of lifting. For example if all the datasets for whose cell-type tree we are building are cell types which share a lineage in development, then we would like to lift as many cell types as possible; however if the dataset considered has only one or two cell types which have a lineage in cell-development then we would expect less lifting. So the value of *α* can be set by the biologist based on biological knowledge, since our method can be used as an exploratory tool. The value of *α* also depends on the length of the data representation, though it is robust over a reasonably wide range of data-representation as shown in Table 4. Now we show that for a fixed length of data representation, we can vary *α* parameters over a reasonably wide range. The results are shown in Table 5. In this experiment we fixed *K*=1000, and simulated these data values for each of the 12-leaf trees and randomly chose lifting paths. We see from the table that even when *α* values vary from 0.1 to 2, the various statistics calculated have stable values. This shows the robustness of the parameters in our method.

**Table 4:** Statistics for trees with different length of data representations

	*K*=500	*K*=1000	*K*=5000
*TPR*	0.783	0.750	0.880
*FPR*	0.088	0.070	0.067
*F*	0.621	0.677	0.733
*ACC*	0.899	0.906	0.927
*RF*	0.700	1.300	0.400

We simulate 10 random 12-leaf trees for varying number of data representation lengths (500, 1000 and 5000) and ran the lifting algorithm (for *α*=0.1). We then calculated the following statistics shown in the table: True positive rate (*TPR*), False positive rate (*FPR*), F-score or *F*_1_-score (*F*), Accuracy (*ACC*), *RF* distance (*RF*)

**Table 5:** Statistics for trees with different values of *α*

	*α*=0.1	*α*=1	*α*=2
*TPR*	0.750	0.750	0.871
*FPR*	0.070	0.063	0.141
*F*	0.677	0.667	0.643
*ACC*	0.906	0.916	0.861

We simulate 10 random 12-leaf trees for data representation length of size 1000 and ran the lifting algorithm for varying values of *α*. We then calculated the following statistics shown in the table: True positive rate (*TPR*), False positive rate (*FPR*), F-score or *F*_1_-score (*F*), Accuracy (*ACC*)

## Conclusions

We proposed a maximum-likelihood based approach to estimate cell-type trees from histone modification data. We showed that our maximum-likelihood based approach outperforms previous approaches such as distance-based or parsimony-based methods, on H3K4me3 histone modification data. We also proposed the first lifting-based approach to infer internal nodes in cell-type trees and showed the usefulness of this technique in both real and simulated data. The lifting approach is important since in cell differentiation, ancestral cell types can coexist with derived cell types in adult organisms. Our approach is easy to use and is probably the only current approach to build cell-type trees with ancestral inference. Hence we feel that our approach will be an effective way to help biologists and bioinformaticians to study the cell differentiation process. The lifting process we developed may also have many other applications, be in the study of cancer genetic data where normal cells differentiate into cancerous cells, and in other diverse fields like the evolution of languages.

## Availability of supporting data

The code for this work can be downloaded from http://lcbb.epfl.ch/software.html.
